# Supplementary material for: Spontaneous Calcium Bursts Organize the Apical Actin Cytoskeleton of Multiciliated Cells
Source: Int J Mol Sci. 2025 Mar 11;26(6):2507. doi: 10.3390/ijms26062507 (PMC11942550; doi:10.3390/ijms26062507)
Supplement: Supplementary file 1 [file ijms-26-02507-s001.zip › Wiegel et al_SupplementalFiguresProof.pdf]

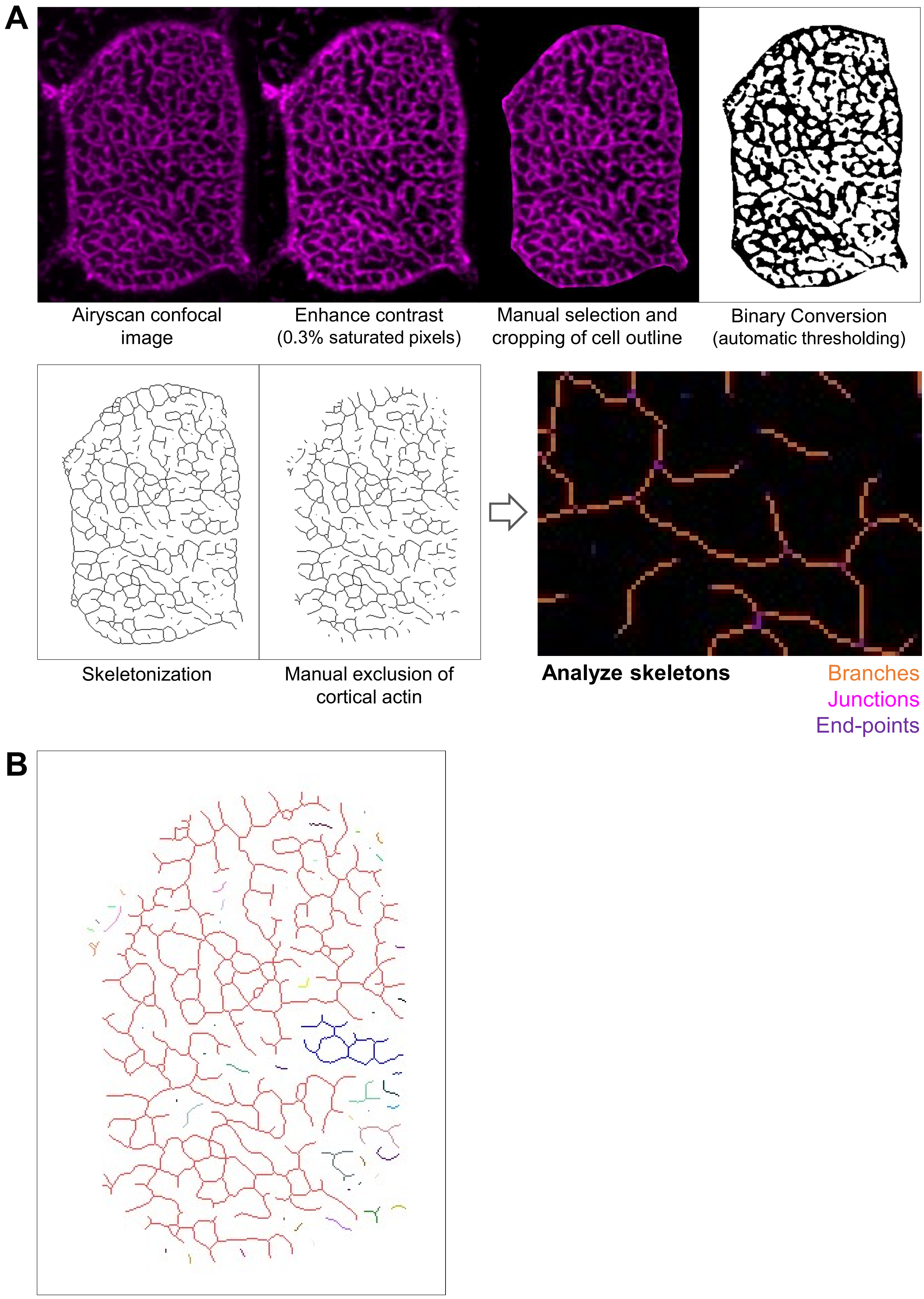

Supplemental Figure S1

Supplemental Figure S1: (A) Procedure of skeletonizing actin in confocal fluorescent images. A skeleton is a distinct, continuous structure of actin, consisting of at least one branch between two end-points. The mature normal apical actin network consists of a low number of highly branched skeletons and a few small fragments. The command “Analyze Skeletons” yields the number of skeletons, branches, junctions and the length of branches. The sum of the length of all branches is the total actin network length. **(B)** Individual actin skeletons labeled in different colors (54 in this examplary cell).

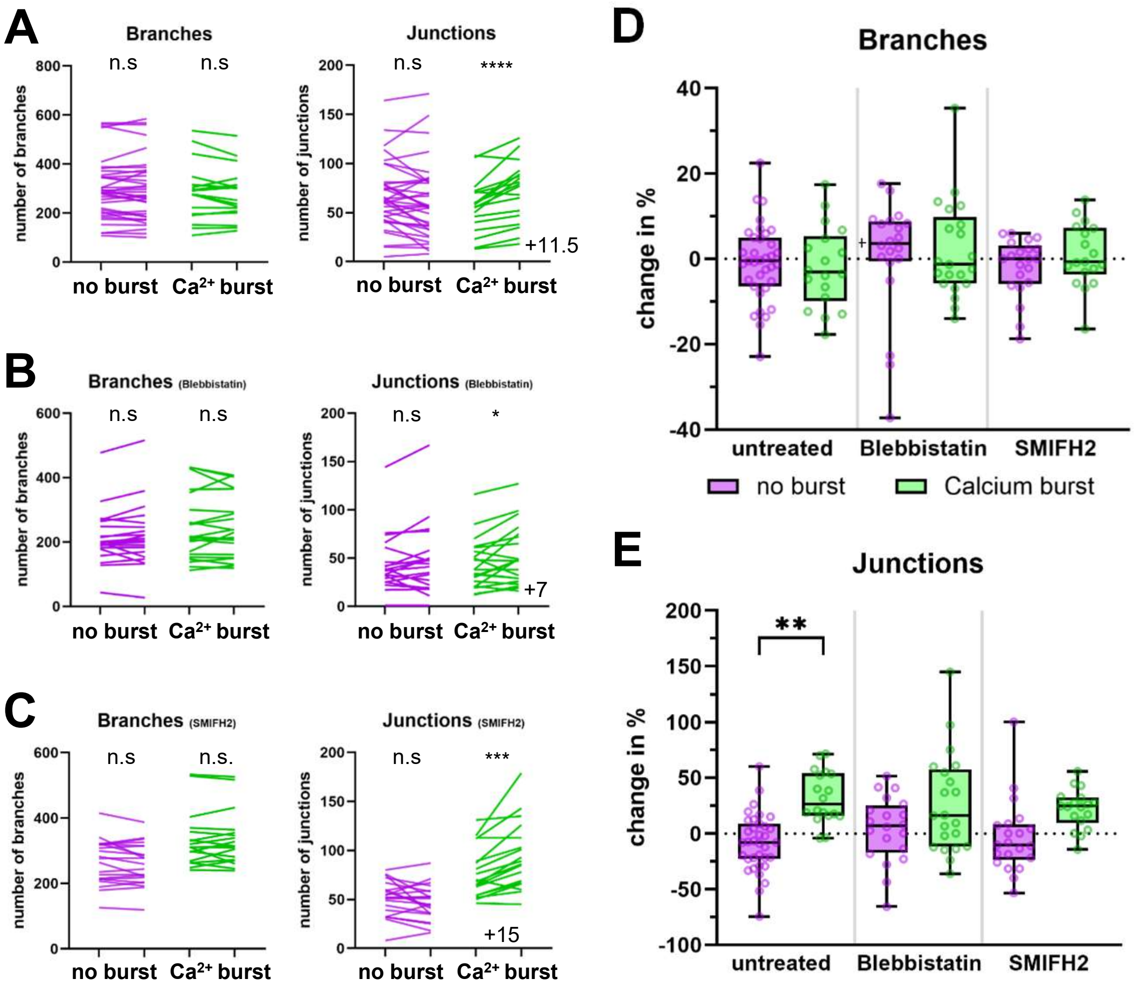

Supplemental Figure S2

Supplemental Figure S2: (A-C) Changes to the actin network (number of branches and junctions) in MCCs with and without calcium burst. (A) untreated embryos, (B) Blebbistatin, (C) SMIFH2. Cells were recorded for 180 seconds and a calcium burst was induced after 12 seconds. Magenta lines show values for individual cells without a calcium burst, green lines for those with an induced calcium burst; end-points indicate the pre- and post-burst values. Statistical test: Wilcoxon matched pairs signed rank test. * $p < 0.05$; ** $p < 0.01$; *** $p < 0.001$; **** $p < 0.0001$; n.s. not significant. For significant changes, the median change is included in the graph. Mean and SD values are provided in table 1. **(D-E)** Comparison of changes to the apical actin network (number of branches, junctions) between MCCs with and without calcium activity for untreated, Blebbistatin-treated, and SMIFH2-treated embryos. Graphs show the changes presented in (A-C) in percent. Each data point represents an individual cell. Box and whiskers indicate quartiles. Averages and SD: Branch number (Untreated, no burst: -0.9±9.1%; calcium burst: -1.9±9.6%; Blebbistatin, no burst: +0.6±13.8%; calcium burst: +2.0±11.4%; SMIFH2, no burst: -1.8±6.9%; calcium burst: +0.8±7.4%). Junction number (Untreated, no burst: -8.2±26.2%; calcium burst: +32.4±23.4%; Blebbistatin, no burst: +3.8±29.7%; calcium burst: +26.1±44.9%; SMIFH2, no burst: -3.7±32.7%; calcium burst: +21.9±17.7). Statistical test: 2-way ANOVA + Tukey's multiple comparisons test. ** $p < 0.01$; non-significant comparisons are not shown. 53 cells from 30 untreated embryos; 41 cells from 14 Blebbistatin-treated embryos; 39 cells from 18 SMIFH2-treated embryos.

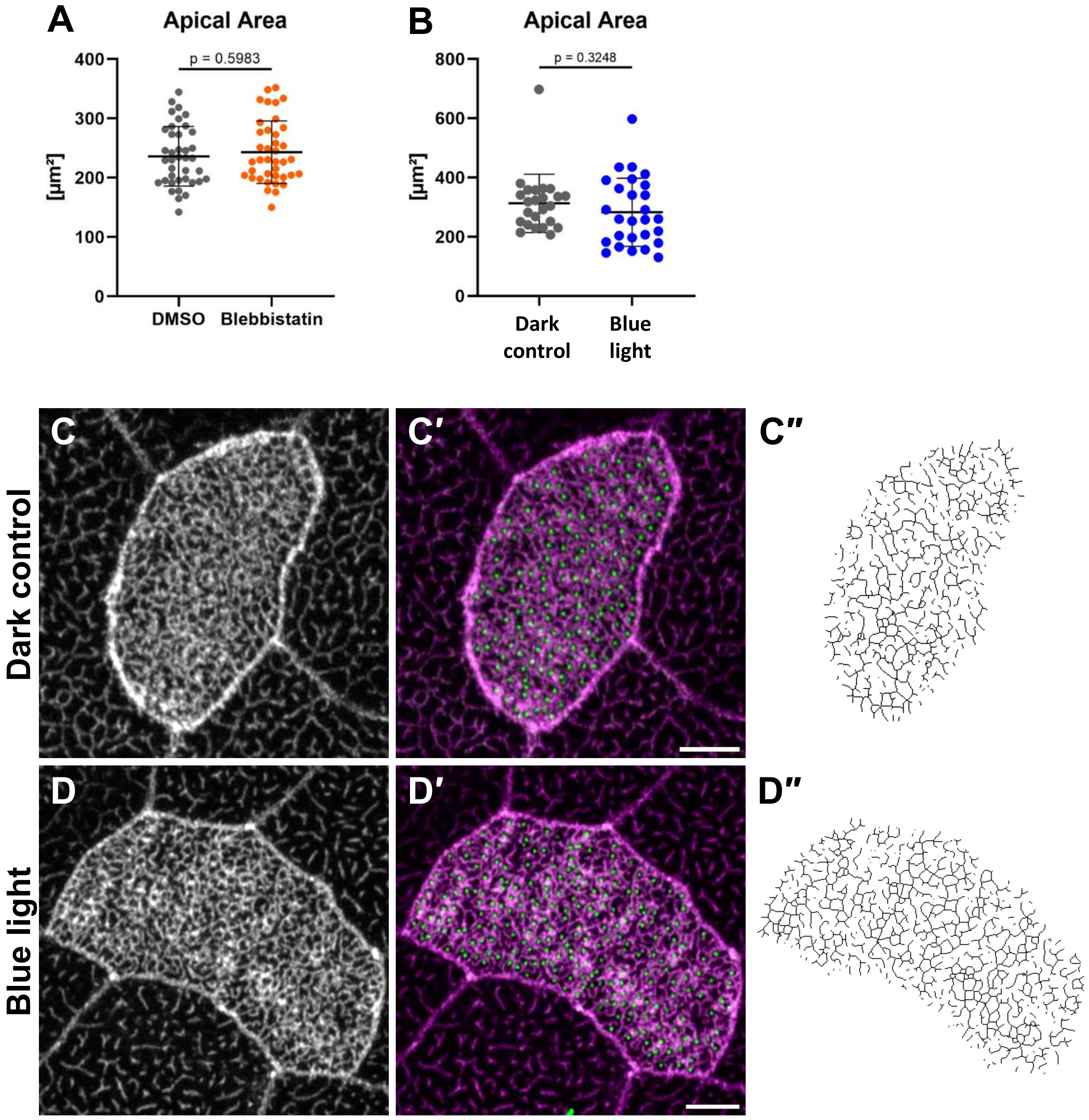

Supplemental Figure S3

Supplemental Figure S3: (A) The apical surface area of MCCs is not changed by Blebbistatin treatment (DMSO 236.1±50.2µm²; Blebbistatin 243.1±52.9µm²). This data belongs to the experiment depicted in Figure 3C. **(B)** The apical surface area of MCCs is not changed by repeated induction of calcium bursts (Dark 312.7±98.2µm²; Blue 282.1±115.0µm²). Statistical test: Mann-Whitney-Test. **(C-D)** Apical actin of MCCs undergoing repeated induction of calcium bursts (D) or controls (C). (C′-D′) Actin labeled by Phalloidin is depicted in magenta, RFP-centrin in green. Scale bars 5µm. (C″-D″) Skeletonized actin network. Data in (B-D) belong to the experiment depicted in Figure 3D-E.

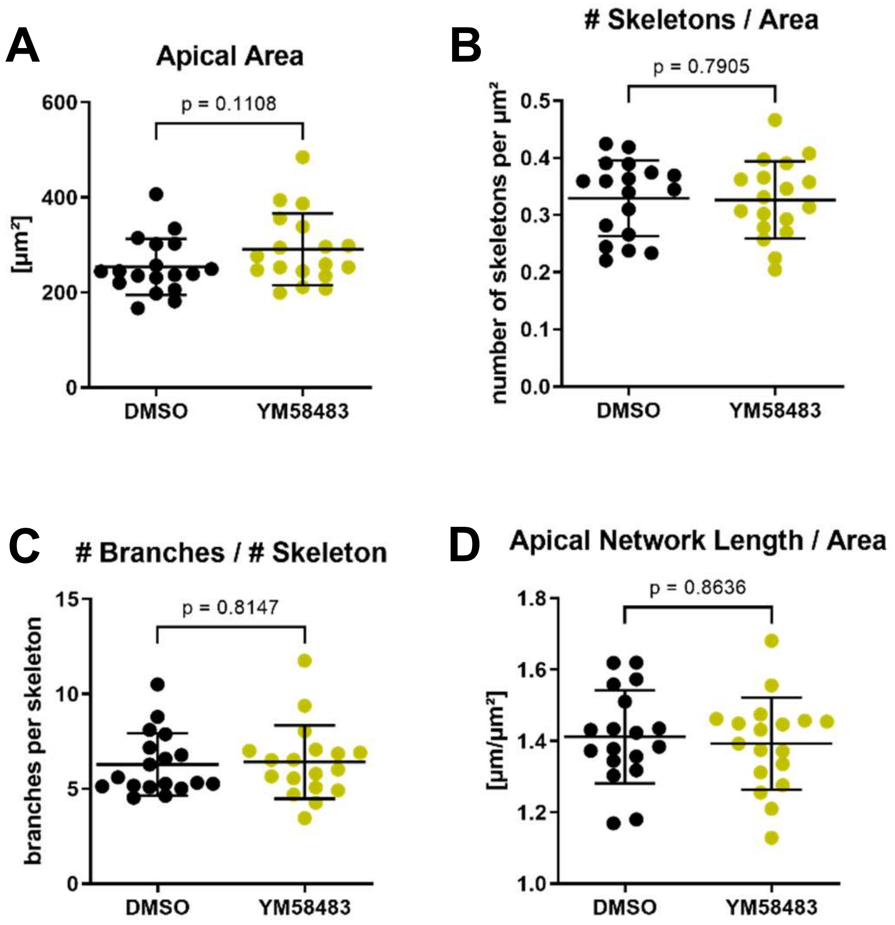

Supplemental Figure S4

Supplemental Figure 4: Quantification of apical surface area and the apical actin network of MCCs in embryos treated with DMSO or YM58483. (A) Apical surface area (DMSO 253.8±59.0μm^2; YM58483 290.9±75.5μm^2). (B) Number of skeletons (separate actin structures) per μm^2 (DMSO 0.33±0.07; YM58483 0.33±0.07). (C) Average branches per skeleton (DMSO 6.3±1.6; YM58483 6.4±1.9). (D) Length of the apical actin network normalized to surface area (DMSO 1.41±0.13μm^{-1}; YM58483 1.39±0.13μm^{-1}). Error bars show mean and SD. 18 cells from 6 embryos analyzed per condition. Statistical test: Mann-Whitney-Test.

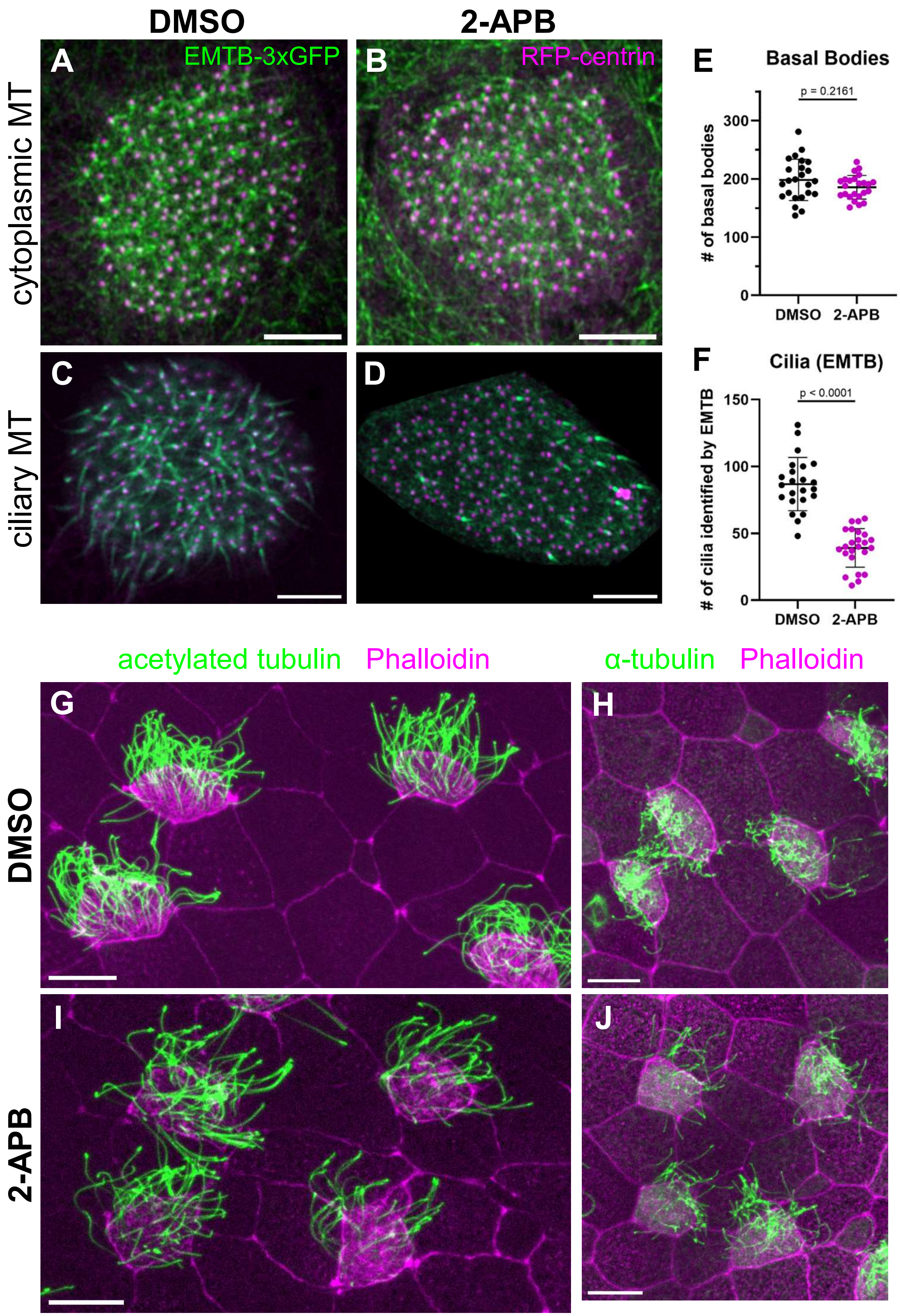

Supplemental Figure S5

Supplemental Figure S5: (A-B) Maximum intensity projections of the apical cytoplasmic microtubular network in MCCs treated with (A) DMSO or (B) 2-APB. **(C-D)** Maximum intensity projections of proximal ciliary microtubules labeled by EMTB-3xGFP (green) in MCCs of embryos treated with (C) DMSO or (B) 2-APB. Basal bodies labeled by RFP-centrin (magenta). Scale bars 5µm **(E)** Basal body number in MCCs treated with DMSO (198±36) or 2-APB (185±20). **(F)** Number of ciliary bases labeled by EMTB-3xGFP in MCCs treated with DMSO (86±20) or 2-APB (39±14). Error bars show mean and SD. 24 cells from 8 embryos analyzed per condition. Statistical test: Mann-Whitney-Test. **(G-J)** Maximum intensity projections of embryonic epidermis stained for Phalloidin (magenta) and (G+I) acetylated tubulin or (H+J) alpha tubulin, respectively (green). Embryos were treated with (G-H) DMSO or (I-J) 2-APB. Scale bars 15µm.

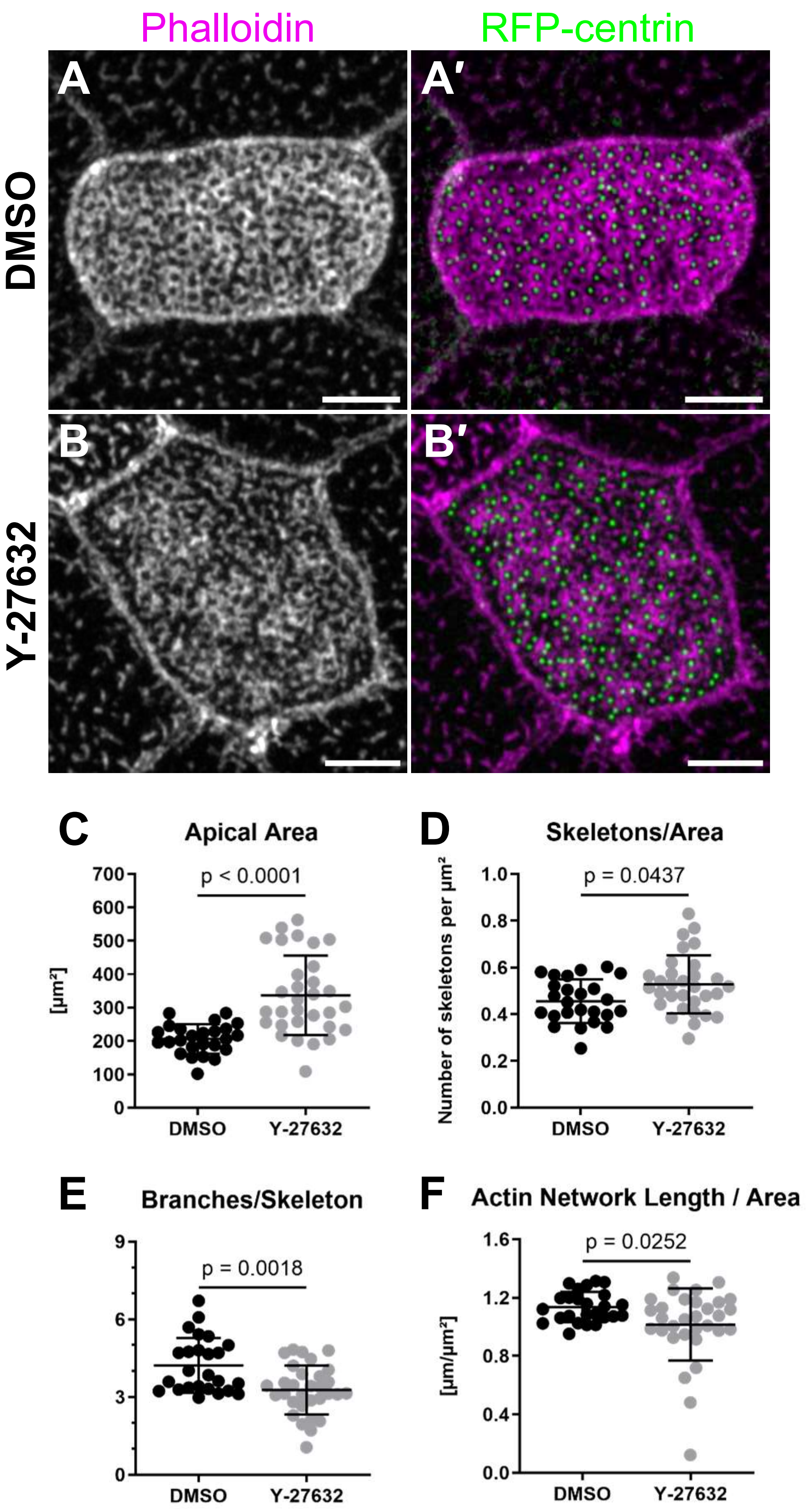

Supplemental Figure S6

Supplemental Figure S6: (A-B) Apical actin in MCCs of embryos treated with (A) DMSO or (B) Y-27632 from stage 19-30. (D′-E′) show actin labeled by phalloidin in magenta, and RFP-centrin labeling basal bodies in green. Scale bars 5µm **(C-F)** Quantification of apical surface area and the apical actin network of MCCs in embryos treated with DMSO or Y-27632. (C) Apical surface area (DMSO 206.1±44.2μm^2; Y-27632 336.5±119.3μm^2). (D) Number of skeletons (separate actin structures) per μm^2 (DMSO 0.46±0.09; Y-27632 0.53±0.12). (E) Average branches per skeleton (DMSO 4.2±1.1; Y-27632 3.3±0.9). (F) Length of the apical actin network normalized to surface area (DMSO 1.14±0.10μm^{-1}; Y-27632 1.02±0.25μm^{-1}). Error bars show mean and SD. 25-31 cells from 8 embryos analyzed per condition. Statistical test: Mann-Whitney-Test.
