# Supplementary material for: Spontaneous Calcium Bursts Organize the Apical Actin Cytoskeleton of Multiciliated Cells
Source: Int J Mol Sci. 2025 Mar 11;26(6):2507. doi: 10.3390/ijms26062507 (PMC11942550; doi:10.3390/ijms26062507)
Supplement: Supplementary file 1 [file ijms-26-02507-s001.zip › Wiegel et al_SupplementalTables.pdf]

Table S1: Mean and SD values from graphs in Figures 2B-D and S2A-C.

untreated	**No burst (first)**	**No burst (last)**	**Pre-burst**	**Post-burst**
**Area**	212.2 ± 81.1μm²	213.9 ± 78.8μm²	236.9 ± 99.1μm²	189.6 ± 80.9μm²
**Skeletons**	186 ± 84	198 ± 86	197 ± 90	148 ± 72
**Branches**	298 ± 123	297 ± 129	288 ± 116	277 ± 104
**Junctions**	66 ± 34	61 ± 38	56 ± 27	71 ± 31
**Avg. branch length**	0.51 ± 0.07μm	0.48 ± 0.07μm	0.48 ± 0.07μm	0.53 ± 0.07μm

Blebbistatin	**No burst (first)**	**No burst (last)**	**Pre-burst**	**Post-burst**
**Area**	164.5 ± 40.9μm²	161.4 ± 38.8μm²	195.0 ± 79.1μm²	181.1 ± 78.1μm²
**Skeletons**	142 ± 43	143 ± 42	175 ± 86	159 ± 81
**Branches**	214 ± 87	220 ± 100	245 ± 103	246 ± 97
**Junctions**	44 ± 30	47 ± 37	44 ± 26	52 ± 30
**Avg. branch length**	0.49 ± 0.09μm	0.47 ± 0.10μm	0.47 ± 0.08μm	0.49 ± 0.07μm

SMIFH2	**No burst (first)**	**No burst (last)**	**Pre-burst**	**Post-burst**
**Area**	188.1 ± 45.8μm²	188.1 ± 47.6μm²	230.1 ± 55.7μm²	187.4 ± 45.9μm²
**Skeletons**	180 ± 51	185 ± 56	195 ± 56	155 ± 43
**Branches**	266 ± 68	259 ± 64	333 ± 84	334 ± 83
**Junctions**	51 ± 18	47 ± 18	77 ± 26	94 ± 35
**Avg. branch length**	0.49 ± 0.07μm	0.48 ± 0.06μm	0.52 ± 0.06μm	0.53 ± 0.05μm
